# The effect of intraoperative lidocaine versus esmolol infusion on postoperative analgesia in laparoscopic cholecystectomy: a randomized clinical trial

**DOI:** 10.1186/s12871-019-0874-8

**Published:** 2019-11-04

**Authors:** Joshan Lal Bajracharya, Asish Subedi, Krishna Pokharel, Balkrishna Bhattarai

**Affiliations:** 1 Department of Anesthesiology & Critical Care, Mechi Zonal Hospital, Bhadrapur, Nepal; 2 Department of Anesthesiology & Critical Care Medicine, BP Koirala Institute of Health Sciences, BPKIHS, Dharan, Nepal; 3 Department of Anesthesiology & Critical Care Medicine, BPKIHS, Dharan, Nepal; 4 Department of Anesthesiology & Critical Care Medicine, BPKIHS, Dharan, Nepal

**Keywords:** Esmolol, Laparoscopic cholecystectomy, Lidocaine, Opioid analgesics, Postoperative pain

## Abstract

**Background:**

As a part of multimodal analgesia for laparoscopic cholecystectomy, both intraoperative lidocaine and esmolol facilitate postoperative analgesia. Our objective was to compare these two emerging strategies that challenge the use of intraoperative opioids. We aimed to assess if intraoperative esmolol infusion is not inferior to lidocaine infusion for opioid consumption after laparoscopic cholecystectomy.

**Methods:**

In this prospective, randomized, double-blind, non-inferiority clinical trial, 90 female patients scheduled for elective laparoscopic cholecystectomy received either intravenous (IV) lidocaine bolus 1.5 mg/kg at induction followed by an infusion (1.5 mg/ kg/h) or IV bolus of esmolol 0.5 mg/kg at induction followed by an infusion (5–15 μg/kg/min) till the end of surgery. Remaining aspect of anesthesia followed a standard protocol apart from no intraoperative opioid supplementation. Postoperatively, patients received either morphine or tramadol IV to maintain visual analogue scale (VAS) scores ≤3. The primary outcome was opioid consumption (in morphine equivalents) during the first 24 postoperative hours. Pain and sedation scores, time to first perception of pain and void, and occurrence of nausea/vomiting were secondary outcomes measured up to 24 h postoperatively.

**Results:**

Two patients in each group were excluded from the analysis. The postoperative median (IQR) morphine equivalent consumption in patients receiving esmolol was 1 (0–1.5) mg compared to 1.5 (1–2) mg in lidocaine group (*p* = 0.27). The median pain scores at various time points were similar between the two groups (*p* > 0.05). More patients receiving lidocaine were sedated in the post-anesthesia care unit (PACU) than those receiving esmolol (*p* < 0.05); however, no difference was detected later.

**Conclusion:**

Infusion of esmolol is not inferior to lidocaine in terms of opioid requirement and pain severity in the first 24 h after surgery. Patients receiving lidocaine were more sedated during their stay in PACU than those receiving esmolol.

**Trial registration:**

ClinicalTrials.gov- NCT02327923. Date of registration: December 31, 2014.

## Introduction

Acute pain after laparoscopic cholecystectomy (LC) is complex in nature, and therefore, opioids alone might not be sufficient to achieve quality analgesia [1, 2]. Besides, usage of only opioids in perioperative settings is associated with undesirable effects [3–6]. In this regard, multimodal regimen (a combination of opioids and non-opioid drug) is recommended for LC, as it provides superior analgesia and improves quality of recovery after surgery [7].

Several strategies using intraoperative intravenous agents have been used for LC to improve the postoperative analgesic profile. Among these, systemic lidocaine is an extensively studied intervention due to its analgesic, anti-hyperalgesic and anti-inflammatory effects [8]. Moreover, doses of IV lidocaine ≤3 mg/kg/h is considered safe and is feasible to use in perioperative setting [8–10]. Surprisingly, the latest Cochrane systematic review demonstrated uncertaininty regarding the beneficial effects of IV perioperative lidocaine on postoperative pain outcomes [11].

In the last decade, intraoperative infusion of the short-acting betablocker esmolol has gained popularity as an alternative technique due to its antinoiceptive and opioid sparing effects [12–14]. A recent meta-analysis has revealed a significant reduction in perioperative opioid consumption with the use of intraoperative esmolol [15].

Although both lidocaine and esmolol are widely used for LC, studies comparing these agents are very few with conflicting results [16, 17]. Therefore, the primary objective of our study was to compare the effects of intraoperative lidocaine and esmolol infusion on postoperative opioid consumption and pain scores following LC. We hypothesized that esmolol infusion would be non-inferior to lidocaine infusion in terms of 24 h postoperative opioid requirement.

## Methods

This prospective, randomized, double-blind, non-inferiority clinical trial was conducted at BP Koirala Institute of Health Sciences between January 2015 and April 2016. The study was approved by the Institutional Ethical Review Board (Ref: IERB 284/014) and the trial was registered prior to patient enrollment at clinicaltrials.gov (NCT02327923). The study was performed according to the Declaration of Helsinki and it adheres to the guidelines of the CONSORT statement.

Female patients aged 18 to 60 years, American society of Anesthesiologist physical status I and II, scheduled for general anesthesia for elective laparoscopic cholecystectomy were enrolled. Exclusion criteria included those with inability to comprehend VAS or severe mental impairment, difficult intubation, pregnancy, morbid obesity, history of epilepsy or allergy to any drugs used in the study, current use of opioids or beta-adrenergic receptor antagonists, baseline heart rate < 50 beats/min, acute cholecystitis, and chronic pain other than cholelithiasis.

Eligible participants were identified during the pre-anesthetic clinic visit. Informed written consent from the recruited patients was taken in the evening before surgery at the in-patient unit. Patients were also instructed about the use of the 10 cm VAS for pain where 0 was “no pain” and 10 was “worst pain”. Oral diazepam (5 mg for ≤50 kg and 10 mg for > 50 kg) was given the night before and 2 h before surgery as premedication.

On the day of surgery at the preoperative holding area, patients were randomly assigned (allocation 1:1) into one of the two groups according to a computer generated random number table. Details of group assignment and case number were kept in a set of sealed opaque envelopes. The anesthesia staff opened the envelope and prepared drugs accordingly. Both the patient and the investigator observing the outcome were blinded to the patient group assignment. The attending anesthesiologist not involved in the study managed the case intraoperatively.

On arrival to the operating room, standard monitoring was applied and baseline heart rate (HR), non-invasive blood pressure, peripheral oxygen saturation and bispectral index (BIS) value (BIS® monitor; Covidien, Boulder, CO, USA) were recorded. General anesthesia was induced with IV fentanyl 1.5 μg/kg and propofol 2–2.5 mg/kg until the cessation of verbal response. Tracheal intubation was facilitated with vecuronium 0.1 mg/kg IV. The lungs were mechanically ventilated using the circle system with 50% mixture of oxygen with air to maintain end tidal carbon dioxide between 35 to 45 mmHg.

During induction, patients in the Lidocaine group received 1.5 mg/kg of lidocaine IV bolus followed by an infusion (Perfusor compact®, B-Braun, Melsungen, Germany) at 1.5 mg/kg/h. Patients in the Esmolol group received an IV bolus of esmolol (0.5 mg/kg) during induction followed by an infusion titrated between 5 and 15 μg/kg/min to maintain the HR within 25% of the baseline value. In both groups, 1 g of IV paracetamol was infused over 15 min after the induction of anesthesia. Anesthesia was maintained with isoflurane targeting mean arterial pressure (MAP) within 20% of baseline, and BIS value between 50 and 60 in both groups. Neuromuscular blockade was maintained with supplemental doses of IV vecuronium after observing the curare notch in capnogram. Hasson’s surgical technique was used. Each port site was infiltrated with 3 ml of 2% lidocaine before incision. Pneumoperitoneum was achieved with carbon dioxide maintaining the intra-abdominal pressure below 15 mmHg. Episodes of intraoperative hypotension (MAP < 65 mmHg) and bradycardia (HR < 50 beats/min) were treated with IV ephedrine 5 mg and atropine 0.4 mg respectively.

No supplemental opioids were used during the surgery. All patients received 30 mg of IV ketorolac after the removal of the gall bladder. At the end of surgery, the carbon dioxide remaining in the peritoneal cavity was expelled by slow abdominal decompression. Both isoflurane and the study drug infusion were discontinued after the skin closure. Incision site was infiltrated with 10 ml of 0.25% bupivacaine. Residual neuromuscular block was reversed with IV neostigmine 0.05 mg/kg and glycopyrrolate 0.01 mg/kg. When the patients were conscious and had adequate muscle power, thorough oropharyngeal suctioning was done and endotracheal tube was removed. The investigator blinded to the group assignment now entered the operating room to collect data on intraoperative hemodynamics side effects. The patients were then transferred to the PACU after they followed verbal commands.

Postoperative pain management included 1 g of paracetamol and 30 mg of ketorolac IV at 6 h and 8 h respectively. The blinded investigator not involved in the anesthesia management assessed VAS pain scores at rest and during movement at the PACU (on arrival, 15 min, 30 min, 1 h) and surgical in-patient-unit (2 h, 6 h, 12 h and 24 h). If the VAS score for pain exceeded > 3 at rest, 1 mg of morphine IV was administered in the PACU, and repeated every five min until the VAS score was ≤3, or if any adverse effects were noticed. These included increased sleepiness (Ramsay sedation scale (RSS) score > 3), respiratory depression (SpO_2_ < 90% in room air or respiratory rate < 8/min). The patients were transferred to the in-patient-unit after 1 h of stay in PACU. In the surgical unit, 50 mg of tramadol IV was administered and further doses of 50 mg was given every 10 min for maintaining VAS score for pain ≤3 (the maximum dose of tramadol was limited to 300 mg in the first 24 h). The tramadol used in surgical unit was converted to morphine equivalent using online calculator (http://clincalc.com/Opioids/).

The primary outcome was opioid consumption (in morphine equivalents) during the first 24 h after surgery. Secondary outcome measures included patient-reported VAS pain scores at rest and movement, postoperative nausea and vomiting (PONV) on a four point scale [18] (1 = no nausea, 2 = mild nausea, 3 = severe nausea, 4 = retching and/or vomiting), the 6-point RSS scores [19](1 = patient anxious and restless, 2 = cooperative and awake, 3 = responding to verbal commands, 4 = responding to mild stimulus, 5 = responding to deep stimulus, 6 = no response). These parameters were noted in PACU and at 2, 6, 12 and 24 h in the surgical unit. PONV grade 3 & 4 were treated with metoclopramide 10 mg IV. Time to first perception of pain and void, overall patient satisfaction from anesthesia at 24 h based on 5-point Likert scale (1 = highly satisfied, 2 = satisfied, 3 = neutral, 4 = dissatisfied, 5 = highly dissatisfied), and occurrence of lidocaine toxicity were also noted. The patients were discharged from the hospital at 24 h after surgery.

Sample size was determined with the aim to reject the inferiority of esmolol infusion compared with lidocaine for the primary outcome of 24 h morphine consumption after surgery. The non-inferiority margin was considered as 2 mg. A sample size of 78 patients (39 per arm) was required to achieve a power of 90%, a one-sided 95% confidence interval, assuming the standard deviation of 3. We finally enrolled 90 patients to allow for possible dropouts or protocol violators (https://www.sealedenvelope.com/power/continuous-noninferior/).

The data collected was entered into excel software and analyzed on STATA version 13.0 (Stata Corporation, College Station, TX, USA). Normality of data was checked using histograms, Skewness-Kurtosis test and Shapiro-Wilk test. Normally distributed data were compared between the two groups using the unpaired Student *t*-test. Mann-Whitney U-tests were used for continuous non-normally distributed data and ordinal data. Comparison of pain scores between the two groups was performed using a mixed effects model. Fixed effects were time of assessment of pain scores postoperatively (15 min to 24 h), study-group assignment (esmolol or lidocaine), and participants in the study as a random effect. Interaction between time of assessment of pain scores and study group was also included in the model and an unstructured covariance matrix was used. For categorical variables, Chi-square test was applied. Time to first perception of pain between the groups was plotted with Kaplan-Meier survival curves and compared with log-rank test. A *p* value < 0.05 was considered as statistically significant.

## Results

**Fig. 1:**
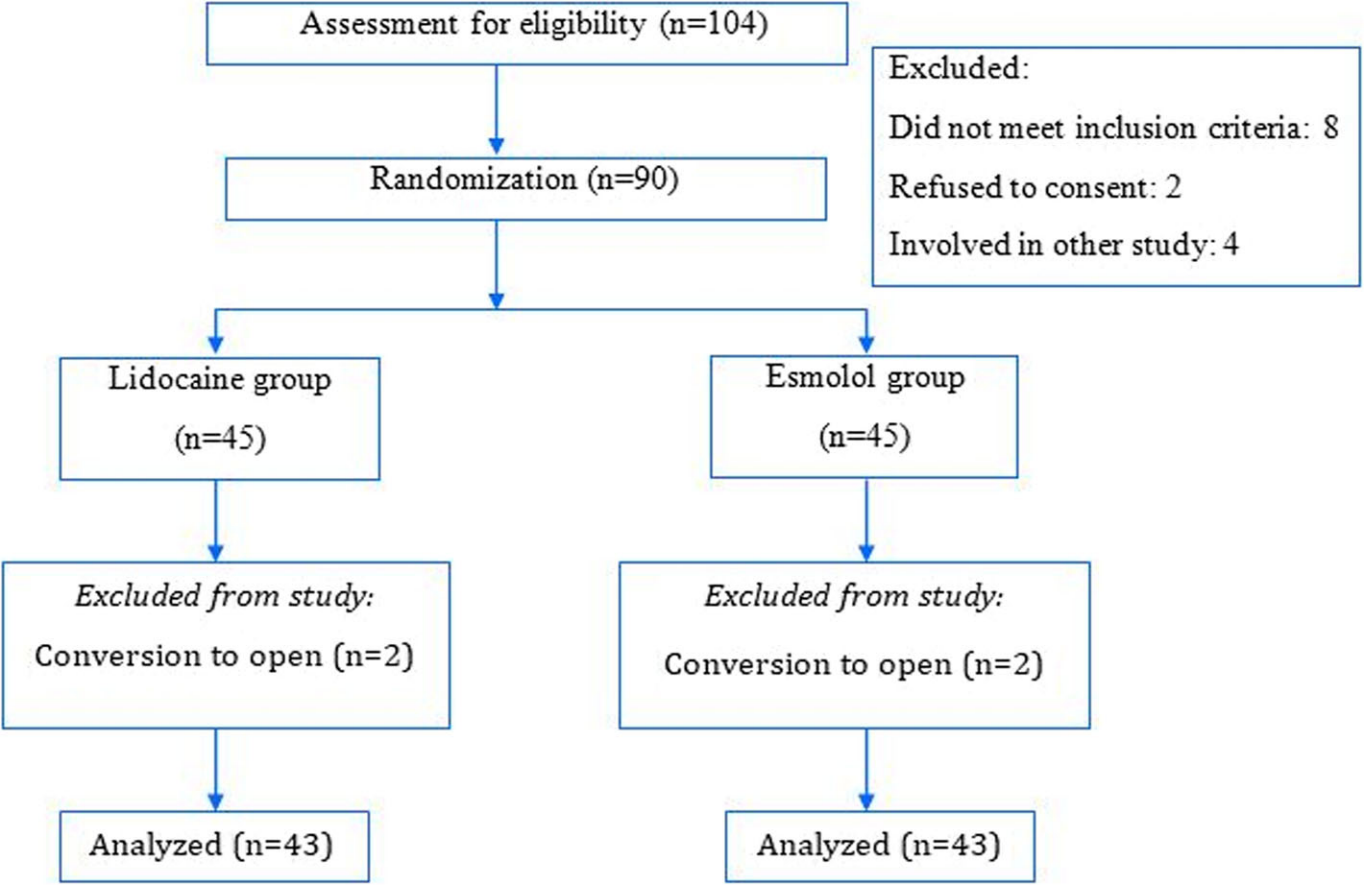
Enrollment, randomization, follow-up and analysis

Among the 104 consecutive patients assessed for eligibility, 90 met the inclusion criteria and they were randomly assigned to lidocaine or esmolol group. Two patients in each group needed conversion to open cholecystectomy, and eventually 86 patients were included in the analysis (Fig. 1). Both the groups were similar with respect to baseline demographic characteristics, duration of surgery and anesthesia time (Table 1).

**Table 1:** Comparison of demographic and baseline characteristics of patients

Variables	Lidocaine group (*n* = 43)	Esmolol group (*n* = 43)
Age (y)	35 (30–49)	40 (27–48)
BMI (kg/m^2^)	22.4 ± 3.1	22.5 ± 2.5
ASA PS I/II	35/8	34/9
Duration of anesthesia (min)	62 (51–77)	57 (48–64)
Duration of surgery (min)	55 (45–75)	50 (45–60)

Values are in median (IQR), mean ± SD, number. Abbreviations: *BMI* body mass index, *ASA PS* American society of Anesthesiologist physical status

**Fig. 2:**
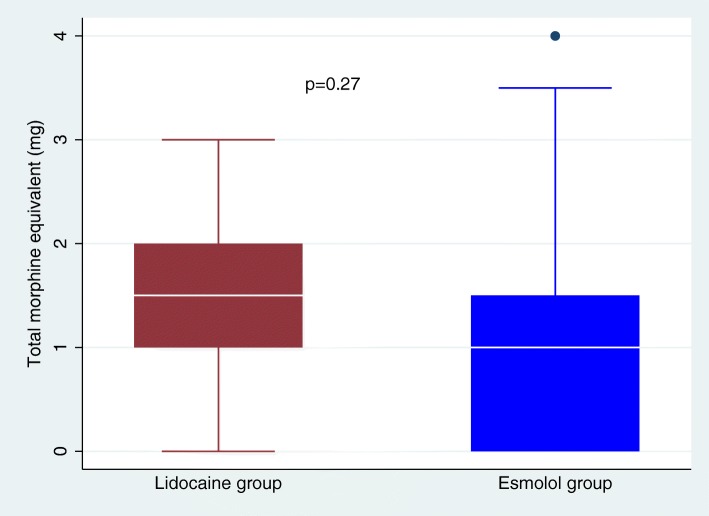
Total 24-h morphine equivalent consumption between the two groups presented as median (IQR)

In the PACU, median morphine consumption was 1 (0–1.5) mg in lidocaine group and 1(0–1.5) mg in esmolol group (*p* = 0.50). Similarly, in the surgical-unit, median tramadol needed was 0 (0–50) mg and 0 (0–50) mg in the lidocaine and esmolol groups, respectively (*p* = 0.65). The median 24 h total morphine equivalent consumed was 1 (0–1.5) mg in the esmolol group and 1.5 (1–2) mg in the lidocaine group (*p* = 0.27; Fig. 2).

**Fig. 3:**
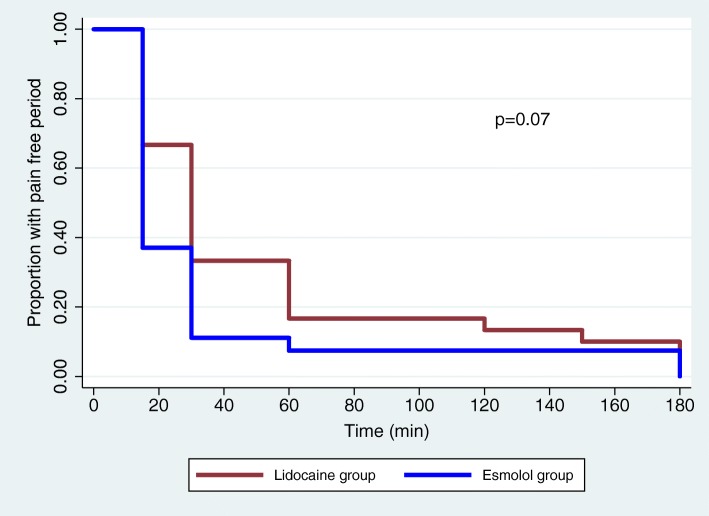
Time to first perception of pain between the two groups

**Fig. 4:**
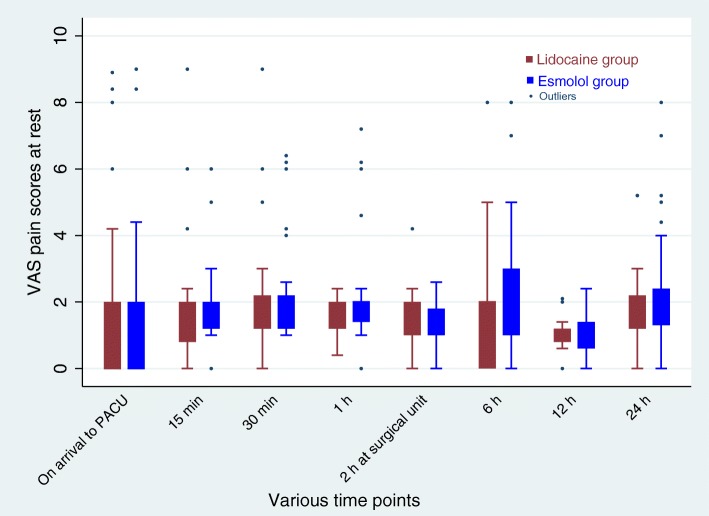
Post-operative pain scores at rest during various time points. Mixed model analysis showed no significant difference in pain scores over times between lidocaine and esmolol group (group time interaction effect, *p* = 0.38). Data are presented as median (IQR)

**Fig. 5:**
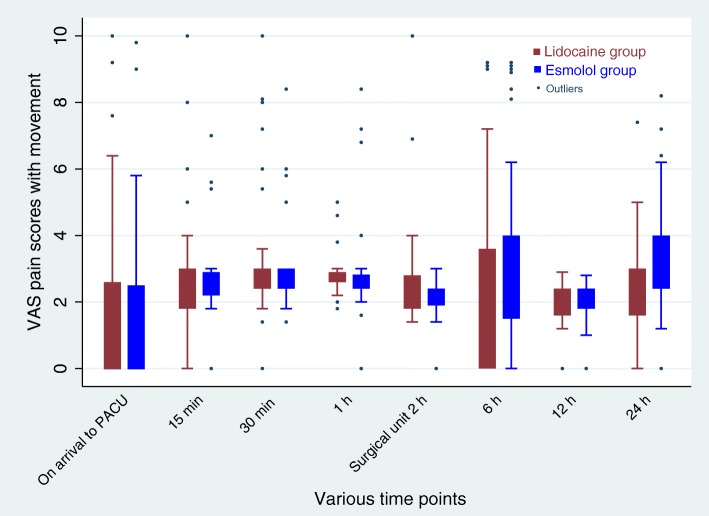
Post-operative pain scores with movement at various time points. Mixed model analysis showed no significant difference in pain scores over times between lidocaine and esmolol group (group time interaction effect, *p* = 0.25). Data are presented as median (IQR)

There was no significant difference for the time until the first perception of pain in the two groups as observed in the survival curve analysis (Fig. 3). Mixed model analysis revealed no difference in postoperative VAS scores for pain at rest (group time interaction effect, *p* = 0.38; Fig. 4) or with movement (group time interaction effect, *p* = 0.25; Fig. 5) between the two groups.

Postoperative sedation scores were comparable except in the PACU where more patients were sedated in lidocaine group (Table 2). Seven patients (16%) in lidocaine had PONV (score ≥ 2) compared to 6 patients (14%) in esmolol group (*p* = 0.71). Median (IQR) satisfaction scores with anesthesia were 2 (2-2) and 2 (2-2) in patients receiving lidocaine and esmolol respectively (*p* = 0.40). The time to first void was similar in the esmolol (2.60 ± 1.2 h) and the lidocaine group (2.67 ± 1.1 h, *p* = 0.79).

**Table 2:** Comparison of postoperative sedation score at various time points

Time point	Lidocaine group (*n* = 43)	Esmolol group (*n* = 43)	*P* value
At PACU			
0 min	5/5/33/0/0/0	4/17/22/0/0/0	0.03
15 min	3/21/19/0/0/0	6/29/8/0/0/0	0.01
30 min	1/32/10/0/0/0	4/36/3/0/0/0	0.01
1 h	1/34/8/0/0/0	1/41/1/0/0/0	0.02
At Surgical unit			
2 h	1/39/3/0/0/0	0/41/2/0/0/0	0.98
6 h	0/41/2/0/0/0	1/40/2/0/0/0	0.66
12 h	0/43/0/0/0/0	0/42/1/0/0/0	0.32
24 h	0/43/0/0/0/0	0/43/0/0/0/0	1

Values are in number of patients with Ramsay sedation scale scores 1/2/3/4/5/6 (1 = patient anxious and restless, 2 = cooperative and awake, 3 = responding to verbal commands, 4 = responding to mild stimulus, 5 = responding to deep stimulus, 6 = no response)

An abdominal drain was inserted in one patient in lidocaine and in 2 patients in esmolol group. Post-hoc analysis revealed that one patient in esmolol group manifested bradycardia intraoperatively, and it responded to IV atropine and pneumoperitoneum decompression. Likewise, one patient in both groups received IV ephedrine 5 mg for hypotensive episode. One patient in esmolol group manifested bronchospasm in PACU and it was managed successfully with salbutamol nebulization. No features of lidocaine toxicity were reported.

## Discussion

This study demonstrated that esmolol is not inferior to lidocaine in terms of postoperative opioid consumption when administered with multimodal analgesia for laparoscopic cholecystectomy. Likewise, pain scores in the first 24 h after surgery was not significantly different between the two groups. The time to first perception of pain, level of satisfaction with anesthesia and any occurrence of side effects were also similar. However, the level of sedation was significantly less in esmolol group than in lidocaine group until 1h in PACU, but no difference was detected thereafter.

Clinical studies investigating the effect of intraoperative IV lidocaine in comparison to esmolol on postoperative opioid and pain scores have shown conflicting results. Similar to our findings, Dogan et al. found no difference between the two groups in postoperative 24 h opioid consumption after laparoscopic cholecystectomy [16]. In contrast, Kavak Akelma et al. found significantly less fentanyl requirement in patients receiving esmolol than those receiving lidocaine infusion or placebo in the first 24 h of surgery [17]. This difference might be due to the higher dose of esmolol (fixed dose, 50 μg/kg/min) used in their patients in comparison to ours (esmolol infusion limited to 15 μg/kg/min). A recent meta-analysis focused on intraoperative use of esmolol on opioid consumption or pain scores found high heterogeneity regarding esmolol dose [15]. Infusion rates varied from 5 to 500 μg/kg/min. Eleven studies had infusion rates ≤15 μg/ kg/min while other 11 studies had infusion rates > 15 μg/kg/min. However, this meta-analysis lacked meta-regression analysis on dose-response relationship. Another meta-analysis exploring the effect of intraoperative esmolol on haemodynamic profiles demonstrated dose-related significant increase in the incidence of hypotension [20]. The authors suggested that frequency of hypotension could be minimized by lowering the initial infusion dose and titrating it according to the hemodynamics response.

As evident from a recent meta-analysis, intraoperative esmolol reduces both intraoperative and postoperative opioid requirement when compared to both remifentanil and non-remifentanil based controls [15]. However, the significant difference in postoperative opioid consumption was limited to the PACU stay only (standard mean difference, − 1.21; 95% CI, − 1.66 to − 0.77). Trials by Dogan et al. [16] and Kavak et al. [17] were not included, and perhaps inclusion of these studies might have further influenced the treatment effects. In a similar model to ours (laparoscopic cholecystectomy) [21], however, with a conventional control consisting of general anesthesia with opioids, the intraoperative fentanyl consumption was 200.5 μg in placebo group while it was null in esmolol group. This reflects that esmolol may have an opioid sparing effect.

Several mechanisms for esmolol antinociceptive effects or opioid-sparing role have been elucidated. These include blockade of the excitatory effects of norepinephrine on pain signals and/or modulation of central adrenergic (pronociceptive) activity [22, 23]. As beta-adrenergic receptors may potentiate the activity of N-methyl-d-aspartate (NMDA) subtype glutamate receptor and facilitate the mechanisms underlying opioid induced hyperalgesia (OIH), beta-adrenergic antagonists are likely to produce antihyperalgesic effects by at least one of these two pathways [24–26]. Clinically, increase in opioid requirement after surgery in patients receiving opioids is likely due to opioid tolerance or OIH, and, as a result it might delay patient’s recovery [21, 27]. Therefore, esmolol may be an effective alternative to counter OIH. Although postoperative opioid sparing effect of esmolol seems promising, the question yet to be answered is whether it is caused directly by its intrinsic properties (anti-nociceptive, antihyperalgesic) and/or indirectly by avoidance of opioids.

Regarding the beneficial role of lidocaine infusion in perioperative setting, the results are confusing. The report from a recent Cochrane based meta-analysis was uncertain if lidocaine had any positive impact on postoperative outcomes [11]. Contrary to this; two other recently published meta-analyses which included only the RCTs comparing lidocaine with placebo in patients undergoing LC found significant reduction in postoperative pain related outcomes in lidocaine group [28, 29]. Perhaps, the use of only placebo comparator in the above mentioned two meta-analyses might have influenced the results. Importantly, there are several reasons for inconsistent results with lidocaine infusion [11], and therefore it is too early to draw a conclusion that perioperative lidocaine infusions are ineffective especially in laparoscopic abdominal surgery.

Early recovery is one of the relevant clinical outcomes after minimally invasive surgery. It is reported that patients in esmolol group achieve early discharge criteria from the PACU as compared to lidocaine group [16]. In the same study, patients receiving lidocaine had RSS scores higher than esmolol at 10 min post-extubation. Likewise, perioperative lidocaine failed to reduce the discharge time after ambulatory surgery compared to placebo when reported as a primary outcome [30]. This is likely due to mild sedative effect of lidocaine and therefore, it could have prolonged the PACU stay. This is in concordance with our results. Patients in the lidocaine group were more sedated up to 1 h after surgery compared to the esmolol group. Although, we did not compare the time to readiness to discharge from the PACU, esmolol has an advantage over lidocaine in relation to discharge time. Moreover, the shorter elimination half-life of esmolol as compared to lidocaine might be beneficial in ambulatory surgery [31, 32].

There are several limitations in our study. First, only female patients were enrolled. There is evidence suggesting that woman experience as well as express more pain after surgery and hence require an excess amount of analgesic agents [33–35]. Hence, sex may be a significant confounding factor in a clinical trial. Although, this difference in pain sensitivity is likely due to biopsychosocial factors/mechanism, laboratory studies investigating sex differences in pain perception are inconsistent [36, 37]. Nevertheless, due to one-gender selected, the external validity of our study may be impaired. Secondly, there was no placebo group and the reason for this is because lidocaine infusion is an effective therapy for laparoscopic procedures and therefore, we used it as an active comparator. Similarly, esmolol has already been shown to reduce opioids requirement after surgery when compared to placebo [15]. Importantly, had we used the placebo group the concern would have been more ethical with no intraoperative opioid supplementation. As evident from a previous study [21], the placebo group required significantly larger doses of opioids intraoperative than in esmolol group. Thirdly, we did not compare the intraoperative hemodynamic parameters. Although, reporting of intraoperative hemodynamic side-effects was not pre-specified, we did post-hoc analysis and found no difference. It is noteworthy that infusions of esmolol and lidocaine at lower doses are safe with no significant alteration in hemodynamics [11, 20]. Finally, the impact of these drugs on readiness to discharge the patients from hospital was not assessed since the patients were required to stay up to 24 h postoperatively following LC in our centre.

It would be interesting to explore the utility of lidocaine and esmolol with adequately powered future comparative studies with regard to PONV, early discharge from the PACU, quality of recovery and length of hospital stay. Also, future studies based on dose-response relationship of esmolol is required that would impact postoperative pain outcomes while lessen the side effects.

## Conclusions

In conclusion, infusion of esmolol is not inferior to lidocaine for postoperative opioid consumption and pain scores following laparoscopic cholecystectomy. However, patients receiving esmolol were less sedated than those receiving lidocaine in the early period after surgery.

## Data Availability

The datasets used and/or analysed during the current study are available from the corresponding author on reasonable request.

## Abbreviations

BIS: Bispectral Index
CONSORT: Consolidated Standards of Reporting Trials
HR: Heart rate
IQR: Interquartile range
IV: Intravenous
MAP: Mean arterial pressure
OIH: Opioid induced hyperalgesia
PACU: Post anesthesia care unit
PONV: Postoperative nausea vomiting
RSS: Ramsay sedation scale
VAS: Visual analogue scale
